# Diagnostic accuracy of menstrual blood for human papillomavirus detection in cervical cancer screening: a systematic review

**DOI:** 10.3332/ecancer.2022.1427

**Published:** 2022-07-14

**Authors:** Priyal Chakravarti, Amita Maheshwari, Shweta Tahlan, Prithviraj Kadam, Sonali Bagal, Suvarna Gore, Nandkumar Panse, Kedar Deodhar, Pankaj Chaturvedi, Rajesh Dikshit, Atul Budukh

**Affiliations:** 1 Centre for Cancer Epidemiology (CCE), Advanced Centre for Treatment, Research and Education in Cancer (ACTREC), Tata Memorial Centre (TMC), Kharghar, Navi Mumbai 410210, Maharashtra, India; 2 Tata Memorial Hospital (TMH), Dr. E Borges Road, Parel, Mumbai 400012, Maharashtra, India; 3 Homi Bhabha National Institute, Training School Complex, Anushaktinagar, Mumbai 400094, India; 4 Homi Bhabha Cancer Hospital, Sangrur 148001, Punjab, India; 5 Rural Cancer Registry, Nargis Dutt Memorial Cancer Hospital, Barshi 413401, Maharashtra, India; a https://orcid.org/0000-0003-2163-796X; b https://orcid.org/0000-0001-7655-1608; c https://orcid.org/0000-0003-1655-684X; d https://orcid.org/0000-0001-6241-9518; e https://orcid.org/0000-0002-2510-1751; f https://orcid.org/0000-0002-2866-5480; g https://orcid.org/0000-0001-6043-3789; h https://orcid.org/0000-0002-6338-8191; i https://orcid.org/0000-0002-3520-1342; j https://orcid.org/0000-0003-4830-0486; k https://orcid.org/0000-0001-6723-802X

**Keywords:** human papillomavirus, menstrual blood, cervical cancer, screening, early detection

## Abstract

Globally, cervical cancer is the fourth most common cancer among females and a major public health problem in low- and middle-income countries (LMICs). There are several screening tests available for cervical cancer screening; however, due to a lack of organised screening facilities as well as factors such as low participation rates in screening programmes, many women die due to cervical cancer. To reach out to a large number of women, an easy, non-invasive and time-saving screening method is required. Evidence supports that cervical cancer screening with human papillomavirus deoxyribonucleic acid (HPV DNA) testing is the most effective technique for lowering the incidence and mortality associated with cervical cancer when compared to other screening methods. Furthermore, a small number of studies have reported that menstrual blood can be used as an alternative sample for HPV detection for cervical cancer screening. We have done a systematic review of the studies that have reported the diagnostic accuracy of menstrual blood to detect HPV. We found five studies in our literature search. The studies showed the diagnostic accuracy of menstrual blood in terms of sensitivity ranging from 82.8% to 97.7% and specificity ranging from 50.0% to 98.0% in cervical intraepithelial neoplasia or HPV infection detection. This review supports the use of menstrual blood as a screening tool for cervical cancer especially in LMICs where women are reluctant to participate in cervical cancer screening due to issues such as embarrassment and discomfort to test procedures as well as busy schedules. However, further studies are required to compare the diagnostic accuracy of menstrual blood in detecting HPV compared to other self-sampled HPV detection methods. This is one of the methods that can be explored further for use as a cervical cancer screening test.

## Introduction

Globally, cervical cancer is the fourth leading cancer in terms of incidence and mortality among females [1]. Of the total 604,127 global cervical cancer cases, 469,036 cases (78%) are from Asian and African countries and 276,647 out of 341,831 (81%) cervical cancer related deaths occurred in these countries [1]. These data highlight the critical necessity of cervical cancer prevention, particularly, in low- and middle-income countries (LMICs). Developed countries have managed to reduce their cervical cancer burden through well-organised screening programmes and infrastructure to provide appropriate follow-up and treatment; however, it is a great challenge for resource-limited countries to organise a national cervical cancer screening programme [2–5]. For LMICs, conducting effective cervical cancer screening necessitates strategic interventions that address not just resource and infrastructural constraints, but should also deal with cultural and geographic factors affecting women’s participation in these screening programmes [5, 6].

Human papillomavirus (HPV) testing, cytology, visual inspection with acetic acid (VIA) and visual inspection with lugol’s iodine are well-known standard screening tests for cervical cancer. There have been significant advancements in cervical cancer prevention techniques. Automated visual evaluation using Artificial Intelligence (AI) is gaining momentum for screening. Also, self-collected cervical samples for HPV deoxyribonucleic acid (HPV DNA) is an upcoming method and undergoing evaluation as a screening test [7–10]. Furthermore, self-sampled HPV testing has opened up an opportunity for improved women’s participation; which is a critical strategy for cervical cancer screening in developing nations [10–14]. However, self-sampled HPV detection faces many challenges in developing countries due to limited resources, high operational cost as well as cultural and socio-economic factors [13, 15]. Most rural women in LMICs work and earn on a daily basis and unfortunately, health is not their utmost priority as they are busy with household and work-related responsibilities. Moreover, due to shy nature, fear of test and social stigma, women are reluctant in participating in screening programmes. To overcome these hurdles, it is required that women are given options for a more convenient and comfortable screening method for HPV detection that allows them to manage their household and work responsibilities.

HPV detection on menstrual blood, although not studied extensively, can be used for HPV based screening. In LMICs, the use of a menstrual cloth/pad is a common menstrual hygiene practice among women and the use of menstrual blood collected on a menstrual pad/cloth as a sample for HPV testing can be an alternative. In most of the African countries as well as countries like India, it has been reported that most of the women use cloth as a menstrual blood absorbent [16–18]. We wanted to analyse the diagnostic accuracy in terms of sensitivity and specificity of the menstrual blood as an additional/alternative sample for HPV testing to reach out for more women in cervical cancer screening programmes. We have found five studies showing the diagnostic accuracy of menstrual blood in HPV detection. The focus of this systematic review is to review the studies that show diagnostic accuracy in terms of sensitivity and specificity of menstrual blood in HPV detection. To the best of authors’ knowledge, this is the first systematic review of these studies.

## Methods

### Eligibility criteria

Studies on the use of menstrual blood to detect HPV were included.

Studies indicating the use of non-invasive menstrual hygiene practices such as menstrual pad/cloth/sanitary napkins to collect menstrual blood were included; studies were excluded if the menstrual hygiene practices included use of tampons or other invasive methods.

Studies showing the diagnostic accuracy results of menstrual blood in HPV or high-risk HPV (HR-HPV) or low-risk HPV (LR-HPV) or cervical intraepithelial neoplasia (CIN) lesions were included. Studies were included if the results were reported in terms of sensitivity and/or specificity.

No restrictions were applied in terms of study designs and study setting due to limited literature search results. We have included studies published up until December 2021. English literature full-text articles published in peer-reviewed journals were included.

### Search strategy

**Figure 1.**
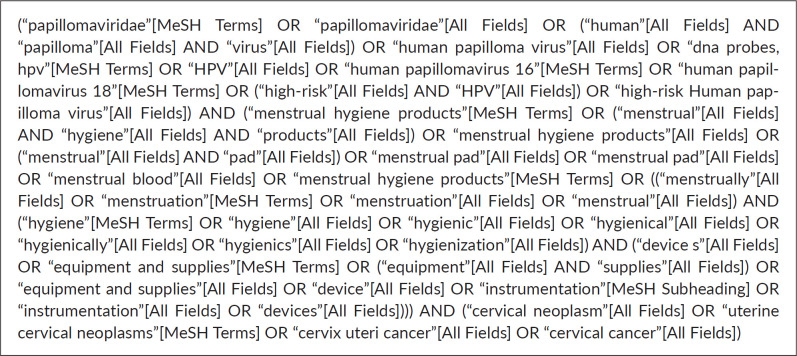
PubMed strategy.

We conducted a thorough search using electronic databases including PubMed, Web of Science and academic search engines like Google Scholar. The potential eligible articles available in the English language were considered. The articles were searched using controlled vocabulary [For PubMed: Medical Subject Headings (MeSH) Terms] as well as keywords including ‘Human Papillomavirus testing’, ‘Menstrual pad’, ‘Cervical cancer screening’, ‘Menstrual Hygiene Products’. The reference list of each eligible article was carefully examined to capture more articles. The search strategy for PubMed is described in Figure 1.

### Study selection

**Figure 2.**
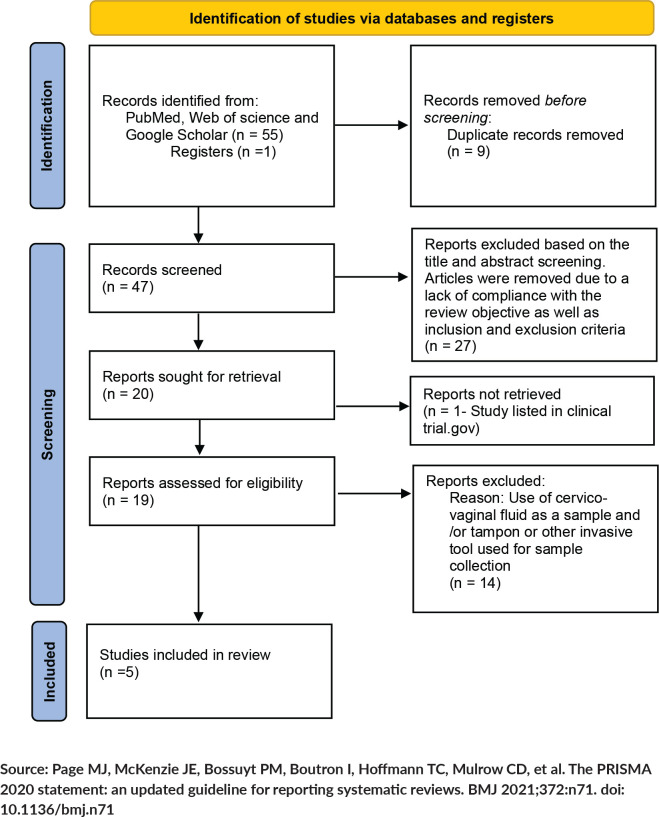
PRISMA 2020 flow diagram.

The number of included and excluded studies at each step is presented in a Preferred Reporting Items for Systematic Review and Meta-Analysis (PRISMA) flow chart (Figure 2).

### Data extraction

Table 1 includes study characteristics such as first author name and publication year, study location, study type, sample specimen, sample collecting tool, menstrual cycle day (MCD), sample size and inclusion criteria, DNA extraction and HPV DNA detection method.

## Results

We identified a total of five studies that met the inclusion criteria of the review [18–22]. The primary aim of these studies was HPV detection using menstrual blood [18–22]; furthermore, few studies also evaluated the prognostic significance of the test [19, 21]. All the studies were conducted in Asian countries including China, Republic of Korea and India. Most of the studies were conducted in a hospital setting such as university hospitals or local clinics [19–22] except one which was at population level [18].

Of these five studies, four studies included women who had a pathological diagnosis of premalignant cervical lesions or positive with HR-HPV infection on cervical sample [19–22]; whereas one study was conducted in a previously unscreened population [18]. The number of participants ranged from 19 to 558 among these studies. Women were asked to provide used menstrual pads in all of the studies [18–22]; however, only one study specified the use of commercially available menstrual pads [22]. A study by Budukh *et al* [18] was done on menstrual pads as well as menstrual cloth as this is a common menstrual hygiene practice in the rural Indian population. In this study, out of the total 557 participants, 527 (94.7%) provided clothes used during menstruation. The study by Lee *et al* [20] used a sanitary napkin with filter attached. Women were asked to provide used sanitary napkins or menstrual pad/cloth in zip-lock plastic bags in all the studies [18–22].

In these five studies, the day on which the menstrual blood was collected ranged from 1 to 4. Budukh *et al* [18] collected menstrual cloth/pad used on MCD 1, Lee *et al* [20] collected menstrual blood on MCD 1 and 2 and Zhang *et al* [22] encouraged patients to provide multiple used sanitary napkins for different MCDs ranging from day 1 to 4. Studies conducted by Wong *et al* [19, 21] did not report the day of sample collection. Furthermore, the sample delivery was mainly by self-delivery to the laboratory [19–22] except in the study conducted by Budukh *et al* [18] where women handed over the used menstrual cloth/pad to health workers who in turn transported the sample to the laboratory.

In the laboratory, a small piece of menstrual pad/cloth was cut by punching [18] or by using sterile scissors [19, 21]; whereas Lee *et al* [20] used attached filter for further investigation. For HPV DNA detection, firstly, DNA extraction was performed using a DNA kit according to the dried blood spot protocol mainly [18, 19, 21, 22] or following the manufacture’s protocol [20]. The details are described in Table 1. The polymerase chain reaction (PCR) method was used for DNA amplification [18–21] except one in which HPV capture hybridisation was used followed by post-capture PCR to amplify the menstrual blood DNA library for sequencing [22]. The primers used in PCR methods were My11 and My09 [19, 21], L1, L2, GP6 and beta-actin [20]. Moreover, targeted region on HPV genome by primers was L1 major capsid gene [19, 21] 185 Base pair (bp) and 108 bp [20]; whereas Zhang *et al* [22] obtained whole-genome sequences of 15 types of HR-HPV were from GenBank and used the Integrated DNA Technologies target capture sequencing to determine HR-HPV genotypes in menstrual blood.

Wong *et al* [19] included 558 women. Of them, 235 women had premalignant cervical disorders including 48 CIN 3, 60 CIN 2, 58 CIN 1 and 69 condyloma acuminatum; whereas, 323 were apparently normal subjects (ANSs). Moreover, from the first cohort of 235 patients, 108 patients with CIN 3 or 2 after Loop Electrosurgical Excision Procedure (LEEP) treatment and 62 patients of CIN 1 or condyloma acuminatum after negative cytology also provided samples. Therefore, there were three cohorts for comparison. Regardless of the menstrual blood HPV DNA test results, all ANSs were referred for a Pap test, and if the Pap test indicated a positive result, a colposcopy with histological confirmation was performed. The results of this study showed that menstrual blood HPV DNA was detected among all 108 high-grade CIN (HGCIN) patients that included 48 CIN 3 and 60 CIN 2 as well as 86 out of 127 (67.7%) patients with low-grade CIN (LGCIN)/condyloma acuminatum. Furthermore, HPV genotyping detected 18 HR-HPV and 11 LR-HPV types. The commonly detected HPV genotypes among menstrual blood of HPV positive subjects before treatment or before complete remission included HR-HPV types HPV 16, 18, 33 and LR-HPV types HPV 6, 81. The HR-HPV was detected in 97 (89.8%) HGCIN cases, 23 (26.7%) LGCIN or condyloma acuminatum cases. Moreover, out of 323 ANSs, menstrual blood HPV DNA was detected in 26 (8.1%) and among these 26 menstrual blood HPV DNA positive ANSs, HR-HPV was detected in 8 (30.8%) samples and LR-HPV in 18 (69.2%) samples. Out of 26 ANSs with menstrual blood HPV DNA positive cases, 2 patients with HR-HPV DNA positive menstrual blood had CIN 1 and another 2 patients with LR-HPV DNA positive had condyloma acuminatum proven by histology, whereas the remaining 22 menstrual blood HPV DNA-positive ANSs (6 HR and 16 LR) had normal cervical cytology. Furthermore, all 297 ANSs with negative menstrual blood HPV DNA had normal cytology. Therefore, the status of those four ANS who had CIN 1 or condyloma acuminatum was changed from ANS to the patient category. After adjustment, the sensitivity, specificity, positive predictive value (PPV) and negative predictive value (NPV) for detection of menstrual blood HPV DNA in samples from patients with CIN or condyloma acuminatum against the reference Pap test were 82.8%, 93.1%, 90.0% and 87.9%, respectively.

Lee *et al* [20] conducted a prospective exploratory pilot study and a total of 19 premenopausal women with high-grade squamous intraepithelial lesion (HSIL) cytology or positivity for HR-HPV on cervical sample within 3 months were included in the study. All participants provided the menstrual blood samples collected on MCD 1 and 2 using a menstrual pad with a filter. Also, the cervical samples were collected for HPV detection by gynaecologists using an AMPLICOR STD swab specimen collection and transport Kit. The final pathologic diagnosis was based on punch biopsy, endocervical curettage, colonisation or hysterectomy. The results showed that the presence of HR-HPV was similar between the cervical and menstrual blood samples. Based on pathology, 9 out of 19 cases were CIN 2+; out of which menstrual blood HR-HPV was detected in eight cases for MCD 1 and 2 combined (MCD 1 only detection = 7/9 and MCD 2 only detection = 6/9). It was found that HR-HPV tests using both cervical and menstrual blood samples have a high sensitivity and NPV, but low specificity and PPV for detecting CIN 2+ lesions. The sensitivity, specificity, PPV and NPV of the menstrual blood HR-HPV tests for detecting CIN 2+ were, respectively, 88.9%, 50.0%, 61.5% and 83.3% during MCD 1 and 2; for MCD 1 samples, these values were 77.8%, 50.0%, 58.3% and 71.4%, respectively; whereas, these were 66.7%, 60.0%, 60.0% and 66.7% for MCD 2 samples. The accuracy rates for detecting HR-HPVs were higher during MCD 1 compared to MCD 2. Comparatively, the sensitivity, specificity, PPV and NPV of the HR-HPV testing using cervical samples for detecting CIN 3+ are 100%, 45.5%, 57.1% and 100%, respectively; and for detecting CIN 2 or worse, 100%, 50.0%, 64.3% and 100%, respectively. Moreover, the genotyping kit detected 40 HPV types including 14 HR-HPV, 7 possible HR-HPV, 14 LR-HPV and 5 undetermined risk HPV.

A population-based study [18] conducted in an unscreened population in Maharashtra state, India included women aged 30–50 years with no history of cervical premalignant or cancer pathology. The study population was divided into population A and population B. Population A included a total of 252 eligible women from two villages; whereas population B included 683 women from 16 villages. In population A, participants were recruited from January 2013 to July 2013. For population B, recruitment was between November 2014 and February 2016. In both groups, women were asked to provide a menstrual pad/cloth used on MCD 1 which was collected by health workers who in turn transported the sample to the laboratory. Furthermore, in population A, all women were requested to undergo Hybrid Capture (HC) 2 testing on cervical sample which was organised by Nargis Dutt Memorial Cancer Hospital. Based on HPV DNA PCR testing done on the menstrual blood sample, 10% of HPV negative cases as well as 18 self-interested women from population B underwent HC2 testing on cervical sample. HPV positive cases based on HPV DNA PCR test done on menstrual blood samples were sent to the hospital for further diagnosis and treatment. Out of 252 eligible women of population A, 192 (76%) women provided menstrual cloth/pad samples and 189 (75%) underwent HC2 testing on cervical sample. Amongst the 192 women who provided a menstrual pad sample, 187 (98%) gave menstrual cloth, whereas 4 (2%) gave a sanitary napkin. A total of 164 (65%) women agreed to both screening tests and were included in the final analysis. Of these 164 women, 6 women tested positive for HPV on menstrual blood HPV testing as well as on HC 2 testing. These six women underwent colposcopic examination and two pre-invasive lesions were diagnosed CIN 2 and CIN 1 one each. The CIN detection rate of 1% was similar in both menstrual cloth/pad HPV DNA test using PCR method and HC2 test. In population B, out of 683 eligible women, 367 (54%) women provided menstrual pad/cloth and DNA amplification through PCR method was performed on 365 samples. Amongst 365 women who provided a menstrual pad, 340 (93.2%) gave menstrual cloth, whereas 25 (6.8%) gave a sanitary napkin. A total of 18 HPV positive cases were identified. Of these 18 cases, only 11 women consented to cervical HPV testing, cervical cytology and colposcopic examination. Additionally, 10% of the randomly selected women with HPV negative on menstrual blood were also subjected to cervical HPV HC2 test. Out of a total of 66 women (11 menstrual blood HPV positive and 55 menstrual blood HPV negative) who underwent HC2 testing, four CIN lesions were identified (one CIN I, two CIN II and one CIN III). For population A and B combined, for the total population, the sensitivity and specificity of menstrual pad HPV testing compared with gold standard HC2 testing were 75.0% and 96.3%. The sensitivity of diagnosing CIN lesion was 83.3% and specificity was 95.1%.

A study by Wong *et al* [21] recruited a total of 265 women diagnosed with CIN or HPV infection as well as 137 ANSs as controls for HPV DNA detection in menstrual blood. Moreover, from the 265 patients, 118 women with CIN 3 or CIN 2 after LEEP and 76 women diagnosed with CIN 1 or HPV infection after complete recovery confirmed by Pap test also provided samples. Therefore, there were three cohorts for comparison. 220 out of 265 women with CIN or HPV infection (83%) were menstrual blood HPV DNA positive, whereas HPV DNA was detected among 6 out of 137 ANSs (2 with HR-HPV type and 4 with LR-HPV types). The menstrual blood HPV positive ANSs further underwent Pap test and results confirmed two menstrual blood HR-HPV positive women had CIN 1 and one out of four menstrual blood HR-HPV positive women had HPV infection. These three women were considered in the patient category and after adjustment, the sensitivity, specificity, PPV and NPV for HPV DNA in the detection of SILs were 83.0%, 98.0%, 99.0% and 74%, respectively. Additionally, the authors conducted a survey to evaluate the acceptance of menstrual blood as a sample for HPV detection by sending a questionnaire to 5,000 women with different socio-demographic backgrounds. Based on the questionnaire responses from 5,000 women, it was noted that women accepted menstrual blood samples for HPV detection. Out of the 5,000 women to whom the questionnaire was sent, 3,700 (74%) participants had undergone a Pap test before, out of which 2,516 (68%) women felt uncomfortable during the procedure due to pain and embarrassment. Further, 4,350 (87%) out of 5,000 women would consider using menstrual blood samples collected on a sanitary napkin for HPV detection and 4,900 (98%) women perceived that this procedure for HPV detection is painless, a time saver and with no embarrassment.

Zhang *et al* [22] evaluated the feasibility and accuracy of HR-HPV detection using menstrual blood HR-HPV capture sequencing. A total of 141 premenopausal women who were HR-HPV positive were recruited in the study; out of which, 120 women were enrolled. Patient recruitment was done from 1 September 2020 to 1 April 2021. The cervical samples were collected from all 120 women for HPV testing by a gynaecologist. In total, 137 sanitary napkins were received from 120 participants. The results of cervical HPV testing indicated 20 patients out of 120 were positive for HPV 16 (16.7%), 12 patients positive for HPV 18 (10.0%) and 93 patients positive for other HR-HPV genotypes (77.5%). The common HR-HPV types found in the samples were HPV 52, HPV 58 and HPV 16. Multiple HR-HPV was detected among 20 patients. A total of 113 patients were tested positive based on the menstrual blood HR-HPV by capture sequencing. Distributions of HPV 16 and HPV 18 versus other HR-HPV genotypes were not significantly different in cervical HPV testing. Overall, as per the results, menstrual blood HR-HPV capture sequencing could find 126 true positive, 3 false positive, 3 false negative and 5 true negative. The sensitivity of menstrual blood testing with capture sequencing was 97.7%. The specificity has not been reported.

Overall, these five studies showed diagnostic accuracy of menstrual blood in terms of sensitivity ranging from 82.8% to 97.7% and specificity ranging from 50% to 98%. Table 2 shows the performance of the menstrual blood sample in detecting HPV that includes reference test, sensitivity, specificity, PPV and NPV.

## Discussion

The screening for precancerous cervical lesions is one of the three objectives set by the World Health Organization (WHO) to achieve the global goal of eliminating cervical cancer by the year 2030. Cervical cancer screening with HPV DNA testing is a proven method. Recent evidence from the International Agency for Research on Cancer suggests that HPV DNA testing is the most effective technique for lowering the cervical cancer incidence and mortality when compared to other screening methods such as cytologic analysis and VIA. It has been observed that the probability of CIN3+ is lower following a negative HPV DNA test result than after a negative cytologic test result over a period of 3–10 years [14].

For self-sampled HPV testing, a variety of samples such as cervico-vaginal fluid or lavages or urine and sample-collection methods including Dacron swabs, cotton-tipped swabs, tampon and cytobrushes have been used [23, 24]. Although many of these methods avoid the personal embarrassment, give flexibility and convenience in terms of time and location, the fact that these sampling devices must be physically placed in one’s genitalia continues to be a cause of concern for some women. Apart from this, administrating the devices correctly for sample collection still concerns women [25]. To overcome these barriers, the few studies have suggested the use of menstrual blood collected on menstrual pad/cloth. Menstrual blood HPV testing is a simple and comfortable approach which provides convenient sample collection for women. To the authors’ knowledge, this is the first systematic review on various studies which included HPV detection through menstrual blood for cervical cancer screening. Overall, five studies conducted in Asian countries evaluated menstrual blood as a potential sample for HPV detection in cervical cancer screening programmes considering its comfort and convenience. These studies reported the sensitivity of menstrual blood ranging from 82.8% to 97.7% and specificity ranging from 50.0% to 98.0%.

HPV detection using menstrual blood has advantages over other patient-performed HPV screening tests. Patient-performed HPV screening tests are not only inconvenient to collect, but also necessitate the storage and delivery of specimens in liquid media; whereas menstrual blood collected on a menstrual pad can simply be placed in a zip-lock bag and mailed to the laboratory. All the studies included in the review used zip-lock plastic bags to store the sample. Menstrual blood samples are both clinically and socially relevant to the ultimate success of primary screening for cervical cancer [18–21].

Menstrual blood used for HPV detection may increase women’s participation in cervical cancer screening as the procedure is non-invasive when using a menstrual pad/cloth, time-saving and convenient. Wong *et al* [21] evaluated the acceptability by women in providing menstrual blood as a sample. The results are promising as out of 5,000 women to whom the questionnaire was sent to assess their willingness in providing a menstrual blood sample, 4,350 (87%) participants would prefer menstrual blood sample for HPV detection compared to traditional Pap test. Non-invasiveness of the test and the comfort of the patient are prime factors while conducting screening for cervical cancer especially in LMICs where many women refuse to take part in the screening programmes due to invasive procedures. A study conducted in a previously unscreened population by Budukh *et al* [18] also supports the acceptability of menstrual pad as a cervical cancer screening tool as it not only eliminates the concern of invasive method but also supports women to manage their daily household activities and as most of the rural women work as daily wage earners they are thus able to save time [18].

It is interesting to note that the delivery of sample by a health worker from participants to the laboratory can be beneficial. It has been reported that the uptake of the screening is increased when samples are collected by a familiar face, such as a health worker [26, 27]. Wong *et al* [21] reported that despite the acceptance of menstrual blood as a sample for HPV testing, out of the total of 5,000 women, 2,200 women (44%) expressed their discomfort regarding mailing a menstrual blood sample to the laboratory. Whereas the study conducted by Budukh *et al* [18] found that women were more likely to participate in the screening and more than 80% compliance for participation was reported where a health worker was stationed to transport the sample. The authors supported and encouraged health workers’ participation in cervical cancer screening, particularly among rural populations. Involvement of health workers may not only overcome barriers like embarrassment of women but can also raise awareness by breaking down stigma and taboos regarding menstrual blood among women, especially in a rural population.

The day of menstrual blood sample collection was documented in three out of five studies and this may also have an impact on the outcome. Based on the results, the authors noted that the viral levels of HR-HPV infections may decrease with the course of menstruation. The authors observed that when menstrual blood HR-HPV tests were performed during MCD 1 in women with CIN 3 or worse, the sensitivity and NPV of the tests, as well as the agreement rates for detecting HR-HPVs using cervical and menstrual blood samples, were at their highest [20]. However, further research is required for standardising the procedure for menstrual blood collection.

Cost effectiveness is one of the most important factors while organising screening especially in LMICs. Organising cervical cancer screening requires a lot of effort as it involves a spacious screening clinic, trained human resources (nurses, medical officer), mobile van and utilisation of the services of village administrative offices. It has been reported that the organising screening clinic costs 20%–40% of the total cost. Menstrual pads used as a cervical cancer screening tool can reduce the cost of organising a screening clinic [18, 28].

Cervical cancer can be prevented easily if we provide easy and comfortable access of diagnosis and treatment to the participants. The major load of this cancer is in LMICs because of the difficulty of organising population based cervical cancer screening programmes. As per World Bank data, the majority of the LMICs population live in rural areas – on average 60% (India: 66%, China: 40%, Sub-Saharan Africa: 59%) [29]. In cervical cancer screening programmes, the screening tool plays an important role. To conduct an effective cervical cancer control programme in LMICs, it is necessary to consider how well a screening tool can be implemented in low-resource settings. The major hurdle in cervical cancer screening is low participation of women and difficult access to the services. Using menstrual devices such as pad/cloth for sample collection provides women with a hassle-free way to participate in cervical screening programmes. This method can lead to increased uptake of screening by women, who can easily participate without leaving their home or work.

The studies showed diagnostic accuracy of menstrual pad in terms of sensitivity in the range of 82.8%–97.7% and specificity 50.0%–98.0%. The diagnostic accuracy values across the studies were comparable. It is interesting to note that all studies have been conducted in Asian countries and the diagnostic performance of this method may vary in different regions of the world. Further studies are required in different parts of the world with social and cultural differences.

The WHO has declared that through cost-effective, evidence-based interventions, we can eliminate cervical cancer by the turn of the century if we achieve the following three goals by 2030 – 90% of girls to receive HPV vaccination by the age of 15 years, 70% of women to undergo screening at least twice in a lifetime and 90% of women with pre-cancer and invasive cancer to receive treatment [30]. The studies have reported that VIA and HPV testing with proper implementation of the programme has potential in preventing the mortality from the cervical cancer [31]. However, due to the COVID-19 pandemic, cervical cancer screening programmes are now facing major hurdles. If 70% of the female population is to be covered in screening, one needs to develop a strategy which can cover the majority of the rural population [32]. We may have to consider an alternative way to improve participation by using self-sampling techniques such as using menstrual cloth/pad to collect menstrual samples for HPV testing and for sample collection using mail services or taking help from health workers. In the COVID-19 pandemic situation, the menstrual blood HPV detection method has an advantage as there is low contact in the screening method and this approach can cover a larger number of women.

The limitation of this review includes the overall smaller sample size as there are only five studies which show low reliability of the results. Furthermore, there is a heterogeneity in the studies such as the sensitivity was checked based on HPV positivity and/or on varying level of cervical neoplasia (from CIN1 to CIN3). Type of menstrual hygiene device used for sample collection may also influence the outcomes in detecting HPV. However, there is no supportive evidence to validate the type of menstrual hygiene devices that should be used for which further research is required. Apart from these limitations, there is a need to compare the menstrual blood HPV detection with other self-sampled HPV detection techniques to validate the method on a large sample size with a standardised method for menstrual blood sample collection, including type of HPV DNA test and the DNA extraction method. Few other factors that require exploration are type of device for menstrual blood collection (pad/cloth) and contamination of sample. Furthermore, HPV testing should be type specific as well as viral region specific (particular areas in the HPV genome are L1, E1/E2 and E6/E7), as per evidence. L1 expression is sometimes lost after HPV integration into the human genome, while E6/E7 expression is always present, which explains why there are no E6- or E7-negative cancers. A test designed solely for L1 will miss around 10% of all invasive tumours [33].

Overall, the use of menstrual blood for HPV detection shows promising results and can be a potential tool for HPV detection, especially in places where cultural and social barriers are more prevalent. This method also holds promise for improved participation of women.

## Conclusion

To conclude, menstrual blood is a potential screening method for under-served women. However, to consider menstrual blood as a sample for HPV detection for a cervical cancer screening strategy by the public health departments of LMICs, further research is required. Initial studies from Asian countries are encouraging; however, larger population-based studies from different parts of the world with social and cultural differences are required.

## Funding

None.

## Conflicts of interest

None.

## Author contributions

Priyal Chakravarti: Literature search, writing manuscript, quality control.

Amita Maheshwari: Conceptualisation, reviewing the draft and providing critical inputs.

Shweta Tahlan: Conceptualisation, reviewing the draft and providing critical inputs.

Prithviraj Kadam: Literature search, assistance in writing the draft, Quality control.

Sonali Bagal: Literature search, assistance in writing the draft, Quality control.

Suvarna Gore: Literature search, assistance in writing the draft, Quality control.

Nandkumar Panse: Reviewing the draft and providing critical inputs.

Kedar Deodhar: Reviewing the draft and providing critical inputs.

Pankaj Chaturvedi: Reviewing the draft and providing critical inputs.

Rajesh Dikshit: Conceptualisation, reviewing the draft and providing critical inputs.

Atul Budukh: Conceptualisation, overall supervision, reviewing the draft and providing critical inputs.

## Figures and Tables

**Table 1.** Studies reported HPV detection using menstrual blood sample.

Study	Wong et al [19]	Lee et al [20]	Budukh et al [18]	Wong et al [21]	Zhang et al [22]
Study location	China	Republic of Korea	India	China	China
Study type	Hospital-based	Hospital-based	Population-based	Hospital-based	Hospital-based
Sample specimen	Menstrual blood	Menstrual blood	Menstrual blood	Menstrual blood	Menstrual blood
Sample collecting tool	Sanitary napkin	Sanitary pad *(pad with a filter attached to the underwear positioned under vaginal orifice)*	Menstrual cloth/sanitary pad	Sanitary pad	Sanitary pad
MCD	Not reported	MCD 1 and 2	MCD 1	Not reported	Multiple MCD ranging from MCD 1 to 5
Sample size and inclusion criteria	**Total participant: 558** **(a) 235** women with pathological diagnosis of CIN, and condyloma acuminatum **1. From this cohort 108** women with CIN 3 and CIN 2 lesions who underwent LEEP **62** CIN 1 and Condyloma acuminatum (CAC) cases with complete remission **(b) 323** sexually active normal subjects in the control group	**Total participants: 19** 19 women with HSILs or HR-HPV infections	**Total participants (who provided menstrual blood sample for HPV): 557** **Population A**: **192** women provided menstrual pad for HPV test **Population B**: **367** women provided menstrual pads for testing and of which **365 samples were processed for DNA** extraction by PCR method. HPV positive cases, 10% randomly selected HPV negative cases underwent HC2 testing	**Total participants: 402** **265** women with pathological diagnosis of CIN or HPV infection **137** Sexually active normal subjects in the control group	**Total participants: 120** **120** women who were premenopausal and had HR-HPV as detected by cervical HPV GenoArray test
DNA extraction	Used QIAamp DNA mini kit as per dried blood spot protocol	Used QIAamp DNA mini kit or LaboPass Tissue Miniprep kit as per manufacture’s protocol	Used QIAamp DNA Micro kit as per dry blood spot protocol	Used QIAamp DNA mini kit as per dried blood spot protocol	Used Tiangen dried blood spot DNA extraction kit
HPV DNA detection method	PCR method	PCR method	PCR method	PCR method	HR-HPV capture sequencing

**Table 2.** Performance of menstrual blood for the detection of HPV infection or CIN lesion.

Study	Wong et al [19]	Lee et al [20]	Budukh et al [18]	Wong et al [21]	Zhang et al [22]
**Reference test**	Pap test and colposcopy with histological confirmation	Histo-pathological test	Cervical HPV DNA test	Pap test and colposcopy with histological confirmation	Cervical HPV test by HPV GenoArray test
**Sensitivity (%)**	82.8 % (in detection of CIN and condyloma acuminatum)	88.9% (CIN 2+ for MCD 1 and 2)	83.3% (in detection of CIN)	83.0% (in detection of CIN or HPV infection)	97.7%
**Specificity (%)**	93.1% (in detection of CIN and condyloma acuminatum)	50.0% (CIN 2+ for MCD 1 and 2)	95.1% (in detection of CIN)	98.0% (in detection of CIN or HPV infection)	Not reported
**PPV^a^**	90.0% (in detection of CIN and condyloma acuminatum)	61.5% (CIN 2+ for MCD 1 and 2)	Not reported	99.0% (in detection of CIN or HPV infection)	Not reported
**NPV^b^**	87.9% (in detection of CIN and condyloma acuminatum)	83.3% (CIN 2+ for MCD 1 and 2)	Not reported	74.0% (in detection of CIN or HPV infection)	Not reported

^a^ PPV, Positive predictive value

^b^ NPV, Negative predictive value
